# Variant near ADAMTS9 Known to Associate with Type 2 Diabetes Is Related to Insulin Resistance in Offspring of Type 2 Diabetes Patients—EUGENE2 Study

**DOI:** 10.1371/journal.pone.0007236

**Published:** 2009-09-30

**Authors:** Trine Welløv Boesgaard, Anette Prior Gjesing, Niels Grarup, Jarno Rutanen, Per-Anders Jansson, Marta Letizia Hribal, Giorgio Sesti, Andreas Fritsche, Norbert Stefan, Harald Staiger, Hans Häring, Ulf Smith, Markku Laakso, Oluf Pedersen, Torben Hansen, for the EUGENE2 Consortium

**Affiliations:** 1 Steno Diabetes Center and Hagedorn Research Institute, Copenhagen, Denmark; 2 Department of Medicine University of Kuopio, Kuopio, Finland; 3 The Lundberg Laboratory for diabetes Research, Department of Internal Medicine, Sahlgrenska University Division of Endocrinology Hospital, Gothenburg, Sweden; 4 Department of Experimental and Clinical Medicine, University Magna Graecia of Catanzaro, Catanzaro, Italy; 5 Department of Internal Medicine, Dialectology, Nephrology, Vascular Medicine and Clinical Chemistry, University of Tubingen, Tubingen, Germany; 6 Faculty of Health Science, University of Aarhus, Aarhus, Denmark; 7 Faculty of Health Science, University of Copenhagen, Copenhagen, Denmark; 8 Faculty of Health Science, University of Southern Denmark, Esbjerg, Denmark; University of Bremen, Germany

## Abstract

**Backround:**

A meta-analysis combining results from three genome-wide association studies and followed by large-scale replication identified six novel type 2 diabetes loci. Subsequent studies of the effect of these variants on estimates of the beta-cell function and insulin sensitivity have been inconclusive. We examined these variants located in or near the *JAZF1* (rs864745), *THADA* (rs7578597), *TSPAN8* (rs7961581), *ADAMTS9* (rs4607103), *NOTCH2* (rs10923931) and the *CDC123/CAMK1D* (rs12779790) genes for associations with measures of pancreatic beta-cell function and insulin sensitivity.

**Methodology/Results:**

Oral and intravenous glucose stimulated insulin release (*n* = 849) and insulin sensitivity (*n* = 596) estimated from a hyperinsulinemic euglycemic clamp were measured in non-diabetic offspring of type 2 diabetic patients from five European populations. Assuming an additive genetic model the diabetes-associated major C-allele of rs4607103 near *ADAMTS9* associated with reduced insulin-stimulated glucose uptake (*p* = 0.002) during a hyperinsulinemic euglycemic clamp. However, following intravenous and oral administration of glucose serum insulin release was increased in individuals with the C-allele (*p* = 0.003 and *p* = 0.01, respectively). A meta-analyse combining clamp and IVGTT data from a total of 905 non-diabetic individuals showed that the C-risk allele associated with decreased insulin sensitivity (*p* = 0.003) and increased insulin release (*p* = 0.002). The major T-allele of the intronic *JAZF1* rs864745 conferring increased diabetes risk was associated with increased 2^nd^ phase serum insulin release during an IVGTT (*p* = 0.03), and an increased fasting serum insulin level (*p* = 0.001). The remaining variants did not show any associations with insulin response, insulin sensitivity or any other measured quantitative traits.

**Conclusion:**

The present studies suggest that the diabetogenic impact of the C-allele of rs4607103 near *ADAMTS9* may in part be mediated through decreased insulin sensitivity of peripheral tissues.

## Introduction

Type 2 diabetes is a multifactorial disease caused by complex interactions between multiple environmental factors and common as well as rare expressions of genetic susceptibility variants. Candidate gene studies and genome wide association (GWA) studies had until early 2008 identified 11 type 2 diabetes loci [1]–[5]. A meta-analysis combining three GWA studies including a total of 10,128 individuals, followed by an extensive replication in 56,975 participants was reported [6]. This study identified six previously unknown loci reaching genome-wide significance levels of association with type 2 diabetes. The most significant variants in the regions were the rs864745 in intron 1 of *JAZF1*, rs12779790 between *CDC123* and *CAMK1D*, rs7961581 between *TSPAN8* and *LGR5*, rs7578597 in exon 24 of *THADA*, rs4607103 near *ADAMTS9* and rs10923931 in intron 5 of *NOTCH2* [6]. Little is known about the function of the encoded gene products in relation to the pathophysiology and molecular pathogenesis of type 2 diabetes. However, three of these variants, *JAZF1* rs864745, *CDC123/CAMK1D* rs12779790, and *TSPAN8* rs7961581, have in a population-based study of 4,516 glucose-tolerant middle aged Danes, shown association with a wide range of measures of insulin release derived from an oral glucose tolerance test (OGTT) reflecting altered beta-cell function [7]. Homozygous carriers of the minor diabetes-associated G-allele of the *CDC123*/*CAMK1D* rs12779790 showed an 18% decrease in insulinogenic index, an 18% decrease in corrected insulin response (CIR), and a 13% decrease in the ratio of area under the serum insulin and plasma glucose curves during an OGTT (AUC-insulin/AUC-glucose) [7]. Carriers of the diabetes-associated T-allele of *JAZF1* rs864745 had an allele dependent 3% decrease in BIGTT-AIR [7]. Furthermore, the diabetes-associated C-allele of *TSPAN8* rs7961581 associated with decreased levels of CIR, AUC-insulin/AUC-glucose ratio, and the insulinogenic index [7]. The remaining three variants did not associate with the OGTT- derived traits or estimates of insulin resistance [7]. Also a study among 1,317 Northern Indians found a reduced insulin secretion following OGTT among individuals carrying the *CDC123/CAMK1D* rs12779790 risk G-allele [8]. These findings were, however, not replicated in a subsequent study among 1,578 German individuals who underwent an OGTT [9]. In the same study the diabetes-related C-allele of *ADAMTS9* rs4607103 associated weakly with insulin resistance when estimated by homeostasis model assessment of insulin resistance (HOMA-IR). Furthermore, a non-significant tendency to association with insulin resistance estimated from a euglycemic-hyperinsulinemic clamp performed in a subgroup of 513 subjects was reported [9]. Given the inconsistency of these previous findings, the aim of the present study was to examine the possible impact of the type 2 diabetes predisposing variants in or near *JAZF1*, *CDC123/CAMK1D*, *TSPAN*/*LGR5*, *THADA*, *ADAMTS9*, and *NOTCH2* loci on pancreatic beta-cell function and insulin sensitivity in non-diabetic European offspring of type 2 diabetic patients, who had been examined with an OGTT and an intravenous glucose-tolerance test (IVGTT). In a subset of 596 of the study participants the insulin sensitivity of peripheral tissues was characterized by applying a euglycemic-hyperinsulinemic clamp.

## Methods

### Subjects

The study included healthy non-diabetic offspring who had one parent with known type 2 diabetes and the other parent with no family history of type 2 diabetes and/or a normal response to an OGTT. The family probands were selected from five white European populations (the EUGENE2 Consortium study populations). Altogether DNA from 849 non-diabetic offspring was available for examination: Danes (*n* = 253), Finns (*n* = 217), Germans (*n* = 149), Italians (*n* = 130), and Swedes (*n* = 100). Clinical and biochemical characteristics of the study population are shown in Table S1. The 149 German offspring included in the present study sample were also part of a published study [9]. The meta-analysis in the present study only included these individuals once.

The participants gave informed written consent, and the study protocols were approved by each center by the local ethical committees: Ethics Committee of the University of Kuopio and Kuopio University Hospital, Finland. Comitato di Bioetica - Azienda Ospedaliera “Mater Domini” & Facoltà di Medicina e Chirurgia, Italy. Ethics Committee of the University of Gothenburg,Sweeden. Ethik-Kommission der Medizinischen Fakultät der Universität Tübingen, Germany and Ethical Committee of Copenhagen, Denmark and in accordance with the principles of the Declaration of Helsinki II.

### Physiology and biochemistry

All study centres followed the same protocol and study participants were examined on two immediate occasions. On the first occasion fasting blood samples were drawn after 12 hours of fasting followed by an OGTT (75 g of glucose) to evaluate glucose tolerance status and OGTT-related serum insulin release; samples for measurements of plasma glucose and serum insulin were drawn at 0, 30, 90, and 120 min during the OGTT. On the second occasion an IVGTT was performed after 12 hours of fasting to determine the first- and second-phase serum insulin release. A bolus of glucose (300 mg/kg in a 50% solution) was given within 30 seconds into the antecubital vein. Blood sampling during IVGTT was done as reported [10]. In a subset of 596 participants the insulin sensitivity of peripheral tissues was measured applying a euglycemic-hyperinsulinemic clamp as detailed [10].

Plasma glucose was measured by the same glucose oxidative method in the different centers and determined by standard laboratory methods [11]. Because serum insulin was measured applying different methods (except for the Gothenburg centre having their insulin measured in Tubingen), the assay applied in Tubingen (micro-particle enzyme immunoassay; Abbott Laboratories, Tokyo, Japan) was selected as a reference assay. Each of the centers in Catanzaro, Copenhagen and Kuopio sent about 100 fasting and post-glucose challenge plasma insulin samples for parallel analysis to the Tubingen laboratory. Serum insulin levels from these three centers were converted by linear regression analysis to serum insulin levels corresponding to the Tubingen assay as previously reported in detail [11], [12]. The first and second phase serum insulin responses were calculated as the area under the serum insulin curve after IVGTT by the trapezoidal method including 0–10 min. and 10–60 min., respectively. Disposition index was calculated as the first phase serum insulin response after IVGTT multiplied by the M value obtained from the euglycemic-hyperinsulinemic clamp. HOMA-IR was calculated as (fasting serum insulin (pmol/l) · fasting plasma glucose (mmol/l))/22.5. Insulinogenic index, the corrected insulin response (CIR), the ratio of area under the serum-insulin and plasma-glucose curves during an OGTT (AUCinsulin/AUC-glucose) and BIGTT-AIR were calculated as previously described [7].

### Anthropometrics

Height and body weight were measured in light indoor clothes and without shoes, and BMI was calculated as weight (kg)/(height(m))^2^. Waist circumference was measured in the upright position midway between the iliac crest and the lower costal margin.

#### Genotyping of DNA isolated from leucocytes

The overall genotyping success rates were >96% for all variants except for *CDC123/CAMK1D* where a 90% genotyping success rate was obtained. When examining 4.5% of the total samples in duplicate the genotyping error rate was 0% for all variants. All genotype distributions obeyed Hardy-Weinberg equilibrium (p>0.05). Genotyping of the polymorphisms were performed applying the TaqMan Allelic Discrimination Assay (Applied Biosystems, Foster City, CA, USA).

### Statistical analyses

Analyses were performed using Statistical Package for Social Science (SPSS Inc., Chicago, IL, USA) version 14.0 and RGui version 2.6.1 (http://www.r-project.org). The results for continuous variables are given as means±SD. A *p*-value<0.05 was considered to be significant. Serum insulin levels were logarithmically transformed prior to statistical analysis. Linear mixed model analysis was applied to adjust for confounding factors. For the linear mixed model analysis centre and pedigree (coded as a family number) were included as random factors, sex as fixed factors, and age, body mass index (BMI) and genotype were added as covariates (applying an additive model). Corrections for multiple testing have not been performed. Based on 95% confidence interval of the estimates of the effect size, we can in the current study exclude effect sizes pr allele above 14% for insulin sensitivity, above 21% for disposition index, above19% for 1^st^ phase insulin secretion and above 17% for 2^nd^ phase insulin secretion including all variants. The meta-analysis was performed using effect size estimates and SE derived from a mixed model (for the present study) and a linear regression analysis (the German study). These effects were based on log10 transformed traits. Both fixed effect (weight of studies estimated using inverse variance) and random effect (weight of studies estimated using DerSimonian-Laird method) meta-analysis were applied [13]. The meta-analysis was adjusted for sex, age and BMI.

## Results

The major C-risk allele of rs4607103 near *ADAMTS9*, conferring increased risk of type 2 diabetes, associated with increased fasting plasma glucose levels (*p* = 0.007) and a reduced insulin-stimulated glucose uptake during a euglycemic-hyperinsulinemic clamp (*p* = 0.002) (Table 1). The C-risk allele also showed statistically significant associations with increased levels of serum insulin at 30 min after oral ingestion of glucose (*p* = 0.01) as well as with increased first and second phase serum insulin release as estimated from an IVGTT (*p* = 0.003 and *p* = 0.009) (Table 1). The *ADAMTS9* rs4607103 C-risk allele did not influence the values of disposition index (Table 1).

**Table 1:** Quantitative- and metabolic-characteristics of 819 non-diabetic offspring stratified according to the genotype of *ADAMTS9* rs4607103.

Genotype	CC	CT	TT	*P_Additive_*
**Quantitative characteristics**				
*n* (men/women)	413 (181/232)	343 (138/205)	63 (26/37)	
Age ( years)	40±9	39±9	39±10	
BMI (kg/m^2^)	26.8±4.9	26.5±5.1	26.1±4.7	0.2
Waist (cm)	90±13	86±14	88±13	0.1
**OGTT**				
**Plasma glucose (mmol/l)**				
Fasting	5.1±0.5	5.0±0.5	4.9±0.5	0.007
30 - min OGTT related	8.3±1.8	8.1±2.0	8.1±2.0	0.3
120 - min OGTT related	6.3±1.6	6.1±1.5	6.1±1.5	0.1
**Serum insulin (pmol/l)**				
Fasting	51±37	50±76	47±30	0.5
30 - min OGTT related	410±266	344±200	356±218	0.01
120 - min OGTT related	352±308	285±216	297±234	0.2
**IVGTT**				
**Serum insulin (pmol/l·min)**				
1^st^ phase insulin secretion	3,618±2,723	3,175±2,596	2,662±1,971	0.003
2^nd^ phase insulin secretion	11,707±11,136	10,296±9,203	7,933±5,815	0.009
**Clamp (** ***n*** ** = 596)**				
M value (umol/kg/min)	39.3±16.3	43.7±16.4	44.2±16.8	0.002
Disposition index (pmol/l·min)x(umol/kg/min)	127,484±122,255	117,791±81,103	104,416±69,374	0.95

Risk allele is denoted in bold. Data are mean±standard deviation. Unadjusted values of serum insulin and derived indices were logarithmically transformed by log10 before statistical analysis. *P*-values were calculated assuming an additive model adjusted for age and sex (BMI and waist), or age, sex, and BMI (all other traits). Indices of insulin release, M value and disposition index were calculated as described in Methods.

**Figure 1:**
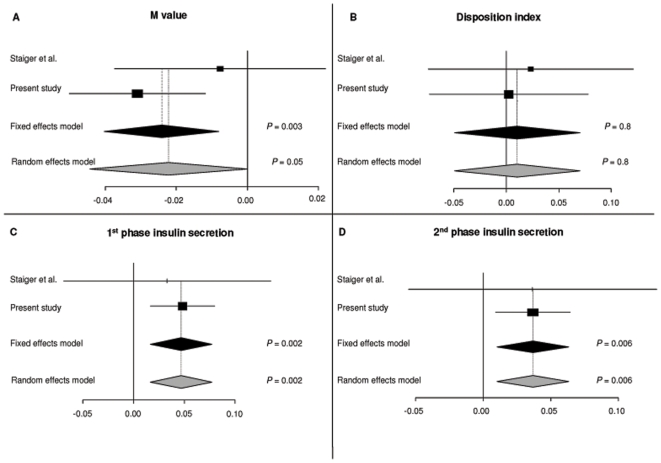
Estimates of effects for the diabetes-associated major C-allele of rs4607103 near *ADAMTS9* (based on log transformed traits and adjusted for sex, age and BMI) of the variants with 95% confidence. A) M value; B) Disposition Index; C) 1^st^ phase insulin secretion and D) 2^nd^ phase insulin secretion. The black diamonds represent the combined and weight (using the DerSimonian-Laird method) effects of the studies. The grey diamonds represent the combined and weight (using inverse variance) effects of the studies.

Clamp and IVGTT derived measures of insulin sensitivity and release (M-value, disposition index, 1^st^ and 2^nd^ phase insulin) were also examined in meta-analyses (Figure 1). The *ADAMTS9* rs4607103 C-allele associated with a decreased M-value in a fixed effect meta-analysis (−0.024, 95%CI [−0.0402;−0.008]) and showed borderline association with decreased M-value in a random effect meta-analysis (−0.022, 95% CI [−0.0443;−0.0001]). The C-allele also associated with increased 1^st^ phase insulin response (0.047, 95%CI [0.017;0.077] (Random) and (0.047, 95%CI [0.017;0.077] (Fixed)) and 2^nd^ phase insulin secretion (0.037, 95%CI [0.011;0.063] (Random) and 0.037, 95%CI [0.011;0.063] (Fixed)). The C-allele did not associate with disposition index in the meta-analysis (0.007, 95%CI [−0.053;0.067] (Random) and 0.037, 95%CI [−0.0153;0.067] (Fixed)). Heterogeneity was examined by Q-test for all traits and none was observed.

The major diabetogenic T-allele of *JAZF1* rs864745 was associated with increased levels of fasting serum insulin (*p* = 0.001) (Table 2). Also, second phase levels of serum insulin following intravenous glucose administration was higher among the T-allele carriers (*p* = 0.03). In the meta-analyse the T-allele also associated with increased 2^nd^ phase insulin secretion (0.03, 95%CI [0.003;0.049] (Random) and 0.03, 95%CI [0.003;0.05] (Fixed) (Table S6). The minor risk allele of the *CDC123/CAMK1D* rs12779790 variant was nominally associated with elevated fasting serum insulin additive model: *p* = 0.07 (Table S4) and recessive model: *p* = 0.05 (adjusted for sex, age, BMI and fasting plasma glucose).

**Table 2:** Quantitative- and metabolic-characteristics of 820 non-diabetic offspring of type 2 diabetes patients, stratified according to genotype of *JAZF1* rs864745.

Genotype	TT	TC	CC	*P_Additiv_*
**Quantitative characteristics**				
*n* (men/women)	244 (95/149)	392 (169/223)	184 (81/103)	
Age±years	38±9	40±10	40±9	
BMI±kg/m^2^	26.4±5.0	26.6±4.8	26.7±5.5	0.8
Waist±cm	88±13	89±13	89±14	0.9
**OGTT**				
**Plasma glucose (mmol/l)**				
Fasting	5.1±0.5	5.1±0.5	5.0±0.5	0.06
30 - min OGTT related	8.1±1.9	8.3±1.9	8.2±1.9	0.6
120 - min OGTT related	6.3±1.5	6.3±1.6	6.1±1.4	0.2
**Serum insulin (pmol/l)**				
Fasting	51±33	53±74	43±29	0.001
30 - min OGTT related	392±258	381±236	360±226	0.09
120 - min OGTT related	344±311	320±289	302±244	0.2
**IVGTT**				
**Serum insulin (pmol/l·min)**				
1^st^ phase insulin secretion	3,253±2,306	3,518±2,812	3,112±2,615	0.2
2^nd^ phase insulin secretion	11,252±9,843	10,931±10,186	9,873±9,939	0.03
**Clamp ** ***n*** ** = 596**				
M value (umol/kg/min)	42±18	41±16	41±16	0.9
Disposition index (pmol/l·min) (umol/kg/min)	113,481±62,700	125,198±90,858	122,397±154,445	0.8

Risk allele is denoted in bold. Data are mean±standard deviation. Unadjusted values of serum insulin and derived indices were logarithmically transformed by log10 before statistical analysis. *P*-values were calculated assuming an additive model adjusted for age and sex (BMI and waist), or age, sex, and BMI (all other traits). Indices of insulin release, M value and disposition index were calculated as described in Methods.

No effect on estimates of serum insulin release or insulin sensitivity was observed for the *NOTCH2* rs10920931, the *TSPAN8* rs7961581, and the *THADA* rs7578597 variants assuming an additive model (Table S2, S3, S4, S5). Similarly, no associations between genotypes and serum insulin release or insulin sensitivity were found when applying recessive and dominant models (data not shown).

Applying additive, dominant and recessive genetic models, respectively, we found no associations to insulinogenic index, the corrected insulin response (CIR), the ratio of area under the serum-insulin and plasma-glucose curves during an OGTT (AUCinsulin/AUC-glucose) and BIGTT-AIR for *CDC123*/*CAMK1D* rs12779790, *JAZF1* rs864745, and *TSPAN8* rs7961581 (data not shown).

## Discussion

In the present study sample of Europeans, enriched in inherited susceptibility to type 2 diabetes, we show that the common diabetes-related C allele of rs4607103 at chromosome 3p14.3-2 upstream of *ADAMTS9* is associated with a decrease in insulin sensitivity of peripheral tissues, as estimated from a euglycemic-hyperinsulinemic clamp. This impairment of insulin sensitivity occurred in the presence of an increase in serum insulin levels in response to intravenous and oral glucose loads. As expected, no difference in disposition index was observed in this non-diabetic high-risk study population of offspring of type 2 diabetes patients. Accordingly, our data indicate that individuals at risk of developing type 2 diabetes at this early stage of disease progression can compensate for the increased insulin resistance by raising insulin release upon glucose stimulation whereas a progressive loss of beta cell function in the presence of progressive insulin resistance will likely over time lead to a decline in glucose tolerance. Thus, our findings suggest that rs4607103 near *ADAMTS9* may after *PPARG* Pro12Ala [14], [15] be the second gene locus conferring risk of type 2 diabetes due to insulin resistance. Interestingly, a recent study of 1,578 Germans reported nominally significant association of the diabetes-related C allele of rs4607103 with an increase in OGTT-derived surrogate measures of insulin resistance [9]. In the same study a subgroup of 513 subjects were examined applying the euglycemic-hyperinsulinemic clamp. Here carriers of the C-allele of rs4607103 showed a trend for having reduced insulin sensitivity [9], a finding which is in line with the present clamp measurements in the larger study population. We performed meta-analyses to estimate the combined effect of *ADAMTS9* rs4607103 on clamp and IVGTT results of insulin sensitivity and release in the two studies. When testing for heterogeneity between the German and the present study in the meta-analyses none was found. Thus the fixed effect model can be applied on these meta-analyses. However, it should be mentioned that the power to detect heterogeneity in meta-analyses including less than 10 studies is low [16]. The meta-analyses support our finding of an association between *ADAMTS9* and decreased insulin sensitivity as well as increased insulin secretion. The lack of a strong effect on insulin sensitivity of rs4607103 in the German study could be explained by the fact that this study population included only 68% with a family history of type 2 diabetes in contract to the present study where all individuals were relatives which mostly likely enriches the inherited susceptibility to type 2 diabetes. The molecular mechanisms behind the effect of ADAMTS9 on peripheral insulin action and risk of diabetes are unknown. ADAMTS9 is a member of the ADAMTS (a disintegrin and metalloproteinase with thrombospondin motifs) protein family which has been implicated in the cleavage of proteoglycans [17], the control of organ maturation and development [18] and inhibition of angiogenesis [19]. *ADAMTS9* is highly expressed in all fetal tissues and in a number of adult tissues where it is abundantly expressed in heart and skeletal muscle [19]. This observed ADAMTS9 expression pattern may be of relevance for our finding of impaired insulin sensitivity of rs4607103 C-allele carriers, as the euglycemic hyperinsulinemic clamp technique primarily measures insulin sensitivity of skeletal muscle. Obviously, prospective studies are needed to establish that a primary diabetogenic effect of *ADAMTS9* rs4607103 is mediated by a decrease in insulin sensitivity of peripheral tissues.

Our study of *JAZF1* rs864745 showed that the major T-allele risk carriers had increased fasting serum insulin levels and slightly elevated 2^nd^ phase insulin secretion after an intravenous glucose load and the meta-analysis support this finding. We did not observe any difference in circulating serum insulin levels after an oral glucose load. In the initial GWA meta-analysis the *JAZF1* variant showed the strongest association with type 2 diabetes [6] and the same variant has subsequently shown association with decreased BIGTT-AIR an estimate of serum insulin release following an OGTT in middle-aged Danes [7]. The reasons for the discrepancy between the previous and the present quantitative trait study are unknown but may be due to ascertainment differences as the previous report is a study of the general population while the current investigation is focused on first degree offspring of type 2 diabetes patients known to be insulin resistant and diabetes-prone. Very little is known about the biological function of JAZF1 but it is established that it is expressed in multiple tissues including pancreas, brain, thalamus, liver, uterus, endometrial and prostate and the gene encodes a nuclear protein with three C2H2-type zinc fingers. The protein functions as a transcriptional repressor of an orphan nuclear receptor (NR2C2) [20] and chromosomal aberrations in the *JAZF1* region associate with endometrial stromal tumors [21].

The remaining four variants; *THADA* rs7578597, *TSPAN8* rs7961581, *NOTCH2* rs10923931 and *CDC123/CAMK1D* rs12779790 did not associate with estimates of serum insulin release or insulin sensitivity in the examined non-diabetic Europeans neither alone or in a meta-analysis of clamp and IVGTT derived traits. This finding may appear to be contrasting to findings in our recent study of 4,516 middle-aged glucose-tolerant individuals of the population-based Inter99 cohort [7]. In the Inter99 study the type 2 diabetes risk alleles in the region between the *CDC123* and the *CAMK1D* and in the *TSPAN8* locus associated with a range of OGTT-based surrogate measures of serum insulin release [7]. The significance of the *CDC123*/*CAMK1D* rs12779790 variant association resisted correction for multiple testing while it was concluded that the association of rs7961581 near *TSPAN8* needed further testing in independent study samples [7]. The reason for non-replication of the Inter99 study findings in the present EUGENE2 study may be related to lack of statistical power to detect the OGTT derived measures of insulin sensitivity and beta-cell function. Based on the effect sizes of the Inter99 study, it was estimated that ∼6,100 and 3,900 subjects, respectively, were needed to achieve 80% statistical power to replicate associations of *CDC123*/*CAMK1D* rs12779790 with insulinogenic index (recessive model), and rs7961581 near *TSPAN8* with insulinogenic index (dominant model) [7].

In conclusion, the present studies of non-diabetic Europeans suggest that the diabetogenic impact of the C-allele of rs4607103 near *ADAMTS9* may in part be mediated through decreased insulin sensitivity of peripheral tissues.

## Supporting Information

Table S1 Clinical characteristics and risk allele frequency in examined non-diabetic individuals according to Eugene2 study centre Data are mean±SD. Risk allele frequency (95%CI) for each risk variant according to Center in Eugene
(0.05 MB DOC)

Table S2 Quantitative- and metabolic-characteristics 820 non-diabetic offspring of type 2 diabetes patients stratified according to genotype of THADA rs7578597 Risk allele is denoted in bold. Data are mean±standard deviation. Unadjusted values of serum insulin and derived indices were logarithmically transformed by log 10 before statistical analysis. P-values were calculated assuming an additive model adjusted for age and sex (BMI and waist), or age, sex, and BMI (all other traits). Indices of insulin release, M value and disposition index were calculated as described in Methods.
(0.05 MB DOC)

Table S3 Quantitative- and metabolic-characteristics 819 non-diabetic offspring of type 2 diabetes patients stratified according to genotype of TSPAN rs7961581 Risk allele is denoted in bold. Data are mean±standard deviation. Unadjusted values of serum insulin and derived indices were logarithmically transformed by log 10 before statistical analysis. P-values were calculated assuming an additive model adjusted for age and sex (BMI and waist), or age, sex, and BMI (all other traits). Indices of insulin release, M value and disposition index were calculated as described in Methods.
(0.05 MB DOC)

Table S4 Quantitative- and metabolic-characteristics 742 non-diabetic offspring of type 2 diabetes patients stratified according to genotype of CDC123/CAMK1D rs12779790. Risk allele is denoted in bold. Data are mean±standard deviation. Unadjusted values of serum insulin and derived indices were logarithmically transformed by log 10 before statistical analysis. P-values were calculated assuming an additive model adjusted for age and sex (BMI and waist), or age, sex, and BMI (all other traits). Indices of insulin release, M value and disposition index were calculated as described in Methods.
(0.05 MB DOC)

Table S5 Quantitative- and metabolic-characteristics 820 non-diabetic offspring of type 2 diabetes patients stratified according to genotype of NOTCH2 rs10923931. Risk allele is denoted in bold. Data are mean±standard deviation. Unadjusted values of serum insulin and derived indices were logarithmically transformed by log 10 before statistical analysis. P-values were calculated assuming an additive model adjusted for age and sex (BMI and waist), or age, sex, and BMI (all other traits). Indices of insulin release, M value and disposition index were calculated as described in Methods.
(0.05 MB DOC)

Table S6 Quantitative traits meta-analysis of associations of SNPs with hyperinsulinaemic euglycemic clamp and IVGTT-derived measurements (M value, Disposition index, 1st and 2nd phase insulin) in the German (n = 330 clamp and n = 103 IVGTT) and the present study (n = 577 clamp and n = 753 IVGTT). Estimates of effects for the diabetogenic risk-allele (based on log 10 transformed traits and adjusted for sex, age and BMI) of the variants with 95% confidence interval.
(0.08 MB DOC)
