# Supplementary material for: Variant near ADAMTS9 Known to Associate with Type 2 Diabetes Is Related to Insulin Resistance in Offspring of Type 2 Diabetes Patients—EUGENE2 Study
Source: PLoS One. 2009 Sep 30;4(9):e7236. doi: 10.1371/journal.pone.0007236 (PMC2747270; doi:10.1371/journal.pone.0007236)
Supplement: Table S1 — Clinical characteristics and risk allele frequency in examined non-diabetic individuals according to Eugene2 study centre Data are mean±SD. Risk allele frequency (95%CI) for each risk variant according to Center in Eugene (0.05 MB DOC) [file pone.0007236.s001.doc]

**Supplementary Table 1. Clinical characteristics and risk allele frequency in examined non-diabetic individuals according to Eugene2 study centre**

|  | **Danish** | **Finnish** | **Swedish** | **Italian** | **German** | **All** |
| --- | --- | --- | --- | --- | --- | --- |
| *n*  (men/women) | 253  (115/138) | 217  (104/113) | 100  (24/76) | 130  (45/85) | 149  (66/83) | 849  (354/495) |
| Age (years) | 40 ± 10 | 35 ± 6 | 40 ± 7 | 44 ± 10 | 44 ± 10 | 39 ± 10 |
| BMI (kg/m^2^) | 26 ± 35 | 26 ± 5 | 25 ± 3 | 28 ± 5 | 28 ± 5 | 27 ± 5 |
| Fasting plasma glucose (mmol/l). | 5.2 ± 0.5 | 5.2 ± 0.4 | 4.8 ± 0.4 | 5.1 ± 0.5 | 5.1 ± 0.5 | 5.1 ± 0.5 |
| Fasting serum insulin (pmol/l) | 44 ± 36 | 44 ± 33 | 45 ± 22 | 54 ± 35 | 54 ± 35 | 50 ± 55 |
| Risk allele frequency % (95%CI) |  |  |  |  |  |  |
| ***ADAMTS9*** rs4607103 | 76.5 (72.8-80.2) | 70.3 (66.0-74.6) | 72.5 (66.3-78.7) | 58.8 (52.2-65.4) | 72.8 (67.8-71.9) | 71.4 (69.2-73.6) |
| ***JAZF1*** rs864745 | 53.1 (48.6-57.5) | 64.1 (49.5-58.8) | 61.0 (54.2-67.8) | 53.2 (46.6-59.8) | 49.3 (43.7-55.0) | 53.7 (51.2-56.1) |
| ***THADA rs7578597*** | 82.2 (85.3-91.0) | 95.6 (93.7-97.5) | 73.0 (66.8-79.2) | 95.0 (92.0-97.9) | 89.9 (86.5-93.3) | 91.5 (90.1-92.8) |
| ***TSPAN rs7961581*** | 26.3 (22.4– 30.2) | 20.5 (16.7-24-3) | 27.0 (20.8-33.2) | 37.6 (31.0-44.0) | 28.5 (23.4-33.7) | 26.7(24.6-28.9) |
| ***CDC123 rs12779790*** | 20.2 (16.6-23.8) | 23.4 (19.1 – 27.7) | 14.7 (4.4-20.0) | 30.0 (24.3-35.7) | 16.3 (11.8-20.8) | 21.3 (19.3-23.4) |
| ***NOTCH2 rs10923931*** | 10.2 (7.5-12.9) | 14.1 (10.8-17.3) | 8.5 (4.6-12.4) | 10.1 (6.1-14.1) | 10.1 (6.7-13.5) | 11.0 (9.5-12.5) |
