# Supplementary material for: Variant near ADAMTS9 Known to Associate with Type 2 Diabetes Is Related to Insulin Resistance in Offspring of Type 2 Diabetes Patients—EUGENE2 Study
Source: PLoS One. 2009 Sep 30;4(9):e7236. doi: 10.1371/journal.pone.0007236 (PMC2747270; doi:10.1371/journal.pone.0007236)
Supplement: Table S2 — Quantitative- and metabolic-characteristics 820 non-diabetic offspring of type 2 diabetes patients stratified according to genotype of THADA rs7578597 Risk allele is denoted in bold. Data are mean±standard deviation. Unadjusted values of serum insulin and derived indices were logarithmically transformed by log 10 before statistical analysis. P-values were calculated assuming an additive model adjusted for age and sex (BMI and waist), or age, sex, and BMI (all other traits). Indices of insulin release, M value and disposition index were calculated as described in Methods. (0.05 MB DOC) [file pone.0007236.s002.doc]

**Supplementary table 2 Quantitative- and metabolic-characteristics 820 non-diabetic offspring of type 2 diabetes patients stratified according to genotype of *THADA* rs7578597.**

| **Genotype** | **TT** | **T**C | CC | ***P_Additiv_*** |
| --- | --- | --- | --- | --- |
| **Quantitative characteristics** |  |  |  |  |
| *n* (men/women) | 683 (293/390) | 134 (51/83) | 3(1/2) |  |
| Age ± years | 39 ± 9 | 39 ± 9 | 53 ± 11 |  |
| BMI ± kg/m^2^ | 26.5 ± 4.9 | 27.0 ± 5.3 | 28.0 ± 2.6 | 0.7 |
| Waist ± cm | 89 ± 13 | 88 ± 14 | 93 ± 5 | 0.6 |
| **OGTT** |  |  |  |  |
| **Plasma glucose (mmol/l)** |  |  |  |  |
| Fasting | 5.1 ± 0.5 | 5.1 ± 0.5 | 5.2 ± 0.3 | 0.9 |
| 30 - min OGTT related | 8.2 ± 1.9 | 8.5 ± 1.7 | 8.2 ± 1.0 | 0.1 |
| 120 - min OGTT related | 6.3 ± 1.5 | 6.1 ± 1.6 | 6.6 ± 1.6 | 0.3 |
| **Serum insulin (pmol/l)** |  |  |  |  |
| Fasting | 49 ± 47 | 56 ± 90 | 40 ± 24 | 0.1 |
| 30 - min OGTT related | 379 ± 243 | 380 ± 227 | 276 ± 263 | 0.6 |
| 120 - min OGTT related | 323 ± 271 | 327 ± 358 | 291 ± 263 | 0.9 |
| **IVGTT** |  |  |  |  |
| **Serum insulin (pmol/l·min)** |  |  |  |  |
| 1^st^ phase insulin secretion | 3,358 ± 2,571 | 3,368 ± 2,935 | 2,808 ± 531 | 0.6 |
| 2^nd^ phase insulin secretion | 10,677 ± 9,908 | 11,469 ± 10,741 | 8,228 ± 3,805 | 0.9 |
| **Clamp *n* = 596** |  |  |  |  |
| M value (umol/kg/min) | 41 ± 17 | 44 ± 17 | 38 ± 11 | 0.1 |
| Disposition index (pmol/l·min) (umol/kg/min) | 120,390 ± 101,917 | 126,039 ± 104,324 | 104,540 ± 25,807 | 0.2 |
