# Supplementary material for: Variant near ADAMTS9 Known to Associate with Type 2 Diabetes Is Related to Insulin Resistance in Offspring of Type 2 Diabetes Patients—EUGENE2 Study
Source: PLoS One. 2009 Sep 30;4(9):e7236. doi: 10.1371/journal.pone.0007236 (PMC2747270; doi:10.1371/journal.pone.0007236)
Supplement: Table S3 — Quantitative- and metabolic-characteristics 819 non-diabetic offspring of type 2 diabetes patients stratified according to genotype of TSPAN rs7961581 Risk allele is denoted in bold. Data are mean±standard deviation. Unadjusted values of serum insulin and derived indices were logarithmically transformed by log 10 before statistical analysis. P-values were calculated assuming an additive model adjusted for age and sex (BMI and waist), or age, sex, and BMI (all other traits). Indices of insulin release, M value and disposition index were calculated as described in Methods. (0.05 MB DOC) [file pone.0007236.s003.doc]

**Supplementary table 3 Quantitative- and metabolic-characteristics 819 non-diabetic offspring of type 2 diabetes patients stratified according to genotype of *TSPAN* rs7961581.**

| **Genotype** | TT | T**C** | **CC** | ***P_Additiv_*** |
| --- | --- | --- | --- | --- |
| **Quantitative characteristics** |  |  |  |  |
| *n* (men/women) | 443 (184/259) | 314 (126/188) | 62 (35/27) |  |
| Age ± years | 39 ± 9 | 40 ± 10 | 39 ± 8 |  |
| BMI ± kg/m^2^ | 26.7 ± 5.1 | 26.6 ± 4.8 | 26.3 ± 3.2 | 0.3 |
| Waist ± cm | 89 ± 13 | 89 ± 13 | 91 ± 13 | 0.9 |
| **OGTT** |  |  |  |  |
| **Plasma glucose (mmol/l)** |  |  |  |  |
| Fasting | 5.1 ± 0.5 | 5.1 ± 0.5 | 5.0 ± 0.5 | 0.6 |
| 30 - min OGTT related | 8.1 ± 1.8 | 8.3 ± 2.0 | 8.7 ± 2.4 | 0.05 |
| 120 - min OGTT related | 6.3 ± 1.5 | 6.2 ± 1.6 | 6.0 ± 1.5 | 0.2 |
| **Serum insulin (pmol/l)** |  |  |  |  |
| Fasting | 48 ± 34 | 50 ± 59 | 44 ± 19 | 0.06 |
| 30 - min OGTT related | 374 ± 223 | 384 ± 262 | 391 ± 250 | 0.3 |
| 120 - min OGTT related | 326 ± 281 | 317 ± 282 | 332 ± 48 | 0.8 |
| **IVGTT** |  |  |  |  |
| **Serum insulin (pmol/l·min)** |  |  |  |  |
| 1^st^ phase insulin secretion | 3,345 ± 2,447 | 3,374 ± 2,753 | 3,723 ± 3,148 | 0.9 |
| 2^nd^ phase insulin secretion | 10,630 ± 8,822 | 10,731 ± 10,765 | 12,288 ± 13,632 | 0.6 |
| **Clamp *n* = 596** |  |  |  |  |
| M value (umol/kg/min) | 40 ± 16 | 43 ± 18 | 38 ± 17 | 0.1 |
| Disposition index (pmol/l·min) (umol/kg/min) | 120,793 ± 111,088 | 115,778 ± 87,429 | 144,679 ± 99,716 | 0.2 |
