# Supplementary material for: Variant near ADAMTS9 Known to Associate with Type 2 Diabetes Is Related to Insulin Resistance in Offspring of Type 2 Diabetes Patients—EUGENE2 Study
Source: PLoS One. 2009 Sep 30;4(9):e7236. doi: 10.1371/journal.pone.0007236 (PMC2747270; doi:10.1371/journal.pone.0007236)
Supplement: Table S4 — Quantitative- and metabolic-characteristics 742 non-diabetic offspring of type 2 diabetes patients stratified according to genotype of CDC123/CAMK1D rs12779790. Risk allele is denoted in bold. Data are mean±standard deviation. Unadjusted values of serum insulin and derived indices were logarithmically transformed by log 10 before statistical analysis. P-values were calculated assuming an additive model adjusted for age and sex (BMI and waist), or age, sex, and BMI (all other traits). Indices of insulin release, M value and disposition index were calculated as described in Methods. (0.05 MB DOC) [file pone.0007236.s004.doc]

**Supplementary table 4 Quantitative- and metabolic-characteristics 742 non-diabetic offspring of type 2 diabetes patients stratified according to genotype of *CDC123/CAMK1D*  rs12779790.**

| **Genotype** | AA | **G**A | **GG** | ***P_Additiv_*** |
| --- | --- | --- | --- | --- |
| **Quantitative characteristics** |  |  |  |  |
| *n* (men/women) | 468 (197/257) | 239 (82/157) | 35 (16/19) |  |
| Age ± years | 39 ± 9 | 39 ± 10 | 38 ± 8 |  |
| BMI ± kg/m^2^ | 26.5 ± 4.7 | 26.8 ± 5.3 | 25.6 ± 4.4 | 0.9 |
| Waist ± cm | 89 ± 13 | 89 ± 13 | 85 ± 12 | 0.5 |
| **OGTT** |  |  |  |  |
| **Plasma glucose (mmol/l)** |  |  |  |  |
| Fasting | 5.1 ± 0.5 | 5.0 ± 0.5 | 5.1 ± 0.5 | 0.7 |
| 30 - min OGTT related | 8.2 ± 1.8 | 8.1 ± 2.0 | 7.9 ± 1.4 | 0.7 |
| 120 - min OGTT related | 6.2 ± 1.6 | 6.2 ± 1.6 | 6.3 ± 1.3 | 0.4 |
| **Serum insulin (pmol/l)** |  |  |  |  |
| Fasting | 51 ± 68 | 48 ± 34 | 36.5 ± 24 | 0.07 |
| 30 - min OGTT related | 383 ± 246 | 372 ± 238 | 308 ± 167 | 0.6 |
| 120 - min OGTT related | 314 ± 287 | 329 ± 294 | 282 ± 168 | 0.7 |
| **IVGTT** |  |  |  |  |
| **Serum insulin (pmol/l·min)** |  |  |  |  |
| 1^st^ phase insulin secretion | 3,316 ± 2,607 | 3,422 ± 2,768 | 3,309 ± 2,734 | 0.9 |
| 2^nd^ phase insulin secretion | 11,204 ± 11,077 | 10,288 ± 9,086 | 8,653 ± 5,511 | 0.3 |
| **Clamp *n* = 596** |  |  |  |  |
| M value (umol/kg/min) | 42 ± 18 | 42 ± 14 | 45 ± 19 | 0.2 |
| Disposition index (pmol/l·min) (umol/kg/min) | 119,660 ± 83,141 | 127,300 ± 137,236 | 130.025 ± 101.528 | 0.3 |
