# Supplementary material for: Variant near ADAMTS9 Known to Associate with Type 2 Diabetes Is Related to Insulin Resistance in Offspring of Type 2 Diabetes Patients—EUGENE2 Study
Source: PLoS One. 2009 Sep 30;4(9):e7236. doi: 10.1371/journal.pone.0007236 (PMC2747270; doi:10.1371/journal.pone.0007236)
Supplement: Table S5 — Quantitative- and metabolic-characteristics 820 non-diabetic offspring of type 2 diabetes patients stratified according to genotype of NOTCH2 rs10923931. Risk allele is denoted in bold. Data are mean±standard deviation. Unadjusted values of serum insulin and derived indices were logarithmically transformed by log 10 before statistical analysis. P-values were calculated assuming an additive model adjusted for age and sex (BMI and waist), or age, sex, and BMI (all other traits). Indices of insulin release, M value and disposition index were calculated as described in Methods. (0.05 MB DOC) [file pone.0007236.s005.doc]

**Supplementary table 5 Quantitative- and metabolic-characteristics 820 non-diabetic offspring of type 2 diabetes patients stratified according to genotype of *NOTCH2* rs10923931**

| **Genotype** | GG | G**T** | **TT** | ***P_Additiv_*** |
| --- | --- | --- | --- | --- |
| **Quantitative characteristics** |  |  |  |  |
| *n* (men/women) | 650 (272/378) | 160 (70/90) | 10 (3/7) |  |
| Age ± years | 39 ± 9 | 41 ± 11 | 35.4 ± 7.5 |  |
| BMI ± kg/m^2^ | 26.6 ± 5.1 | 26.7 ± 4.6 | 26.9 ± 3.2 | 0.7 |
| Waist ± cm | 89 ± 14 | 90 ± 13 | 88 ± 14 | 0.8 |
| **OGTT** |  |  |  |  |
| **Plasma glucose (mmol/l)** |  |  |  |  |
| Fasting | 5.1 ± 0.5 | 5.1 ± 0.5 | 5.3 ± 0.5 | 0.2 |
| 30 - min OGTT related | 8.2 ± 1.9 | 8.4 ± 2.0 | 8.0 ± 0.9 | 0.3 |
| 120 - min OGTT related | 6.2 ± 1.5 | 6.4 ± 1.7 | 6.4 ± 0.9 | 0.2 |
| **Serum insulin (pmol/l)** |  |  |  |  |
| Fasting | 49 ± 50 | 55 ± 78 | 44 ± 19 | 0.6 |
| 30 - min OGTT related | 374 ± 240 | 399 ± 244 | 371 ± 227 | 0.3 |
| 120 - min OGTT related | 315 ± 280 | 355 ± 319 | 323 ± 138 | 0.2 |
| **IVGTT** |  |  |  |  |
| **Serum insulin (pmol/l·min)** |  |  |  |  |
| 1^st^ phase insulin secretion | 3,315 ± 2,591 | 3,476 ± 2,824 | 3,493 ± 1,190 | 0.1 |
| 2^nd^ phase insulin secretion | 10,726 ± 9,972 | 11,066 ± 10,510 | 11,152 ± 9,673 | 0.1 |
| **Clamp *n* = 596** |  |  |  |  |
| M value (umol/kg/min) | 42 ± 17 | 41 ± 16 | 38 ± 17 | 0.6 |
| Disposition index (pmol/l·min) (umol/kg/min) | 120,439 ± 107,501 | 123,297 ± 80,209 | 121,894 ± 42,084 | 0.5 |
