# Supplementary material for: Variant near ADAMTS9 Known to Associate with Type 2 Diabetes Is Related to Insulin Resistance in Offspring of Type 2 Diabetes Patients—EUGENE2 Study
Source: PLoS One. 2009 Sep 30;4(9):e7236. doi: 10.1371/journal.pone.0007236 (PMC2747270; doi:10.1371/journal.pone.0007236)
Supplement: Table S6 — Quantitative traits meta-analysis of associations of SNPs with hyperinsulinaemic euglycemic clamp and IVGTT-derived measurements (M value, Disposition index, 1st and 2nd phase insulin) in the German (n = 330 clamp and n = 103 IVGTT) and the present study (n = 577 clamp and n = 753 IVGTT). Estimates of effects for the diabetogenic risk-allele (based on log 10 transformed traits and adjusted for sex, age and BMI) of the variants with 95% confidence interval. (0.08 MB DOC) [file pone.0007236.s006.doc]

Supplementary table 6 Quantitative traits meta-analysis of associations of *SNPs* with hyperinsulinaemic euglycemic clamp and IVGTT-derived measurements (M value, Disposition index, 1^st^ and 2^nd^ phase insulin) in the German (*n*=330 clamp and *n*=103 IVGTT) and the present study (*n*=577 clamp and *n*=753 IVGTT)

| **SNP/**  **Diabetogenic risk-allele Trait** | **Fixed model**  **(95%CI)** | ***P***  **value** | **Random effect**  **(95%CI)** | ***P* value** | ***P***  **test of heterogeneity** |
| --- | --- | --- | --- | --- | --- |
| *JAZF1* **rs864745/**  Major T risk-allele |  |  |  |  |  |
| M  value (umol/kg/min) | 0.003  (-0.011; 0.017) | 0.7 | 0.003  (-0.017; 0.018) | 0.7 | 0.7 |
| 1^st^ phase insulin secretion  pmol/min | 0.028  (-0.048; 0.104) | 0.5 | 0.028  (-0.048; 0.104) | 0.5 | 0.3 |
| 2^nd^ phase insulin secretion  pmol/min | 0.026  (0.003; 0.049) | 0.03 | 0.026  (0.003; 0.049) | 0.03 | 0.9 |
| Disposition index  (pmol/l·min) (umol/kg/min) | 0.010  (-0.411; 0.060) | 0.7 | 0.010  (-0.041; 0.060) | 0.7 | 0.98 |
| *NOTCH2* **rs10923931/**  Minor T risk-allele |  |  |  |  |  |
| M value (umol/kg/min) | -0.001  (-0.026; 0.024) | 0.9 | 0.0007  (-0.029; 0.030 | 0.9 | 0.3 |
| 1^st^ phase insulin secretion  pmol/min | 0.036  (-0.009; 0.081) | 0.1 | 0.036  (-0.009; 0.081) | 0.1 | 0.8 |
| 2^nd^ phase insulin secretion  pmol/min | 0.033  (-0.006; 0.072) | 0.1 | 0.033  (-0.006; 0.072) | 0.1 | 0.9 |
| Disposition index  (pmol/l·min) (umol/kg/min) | 0.04  (-0.055; 0.135) | 0.4 | 0.04  (-0.055; 0.135) | 0.4 | 0.9 |
| *THADA* **rs7578597/**  Major T risk-allele |  |  |  |  |  |
| M value (umol/kg/min) | -0.011  -0.414; 0.019) | 0.5 | 0.0006  (-0.061; 0.063) | 0.98 | 0.08 |
| 1^st^ phase insulin secretion  pmol/min | -0.029  (-0.205; 0.147) | 0.7 | -0.029  (-0.205; 0.147) | 0.7 | 0.8 |
| 2^nd^ phase insulin secretion  pmol/min | -0.006  (-0.050; 0.037) | 0.8 | -0.009  (-0.064; 0.045) | 0.7 | 0.3 |
| Disposition index  (pmol/l·min) (umol/kg/min) | -0.039  (-0.147; 0.068) | 0.5 | -0.035  (-0.159; 0.088) | 0.6 | 0.3 |
| *TSPAN* **rs7961581/**  Minor C risk-allele |  |  |  |  |  |
| M value (umol/kg/min) | 0.002  (-0.014: 0.018) | 0.8 | -0.003  (-0.040; 0.034) | 0.9 | 0.03 |
| 1^st^ phase insulin secretion  pmol/min | 0.0028  (-0.026; 0.032) | 0.8 | 0.003  (-0.026; 0.032) | 0.8 | 0.8 |
| 2^nd^ phase insulin secretion  pmol/min | 0.006  (-0.020; 0.031) | 0.7 | 0.006  (-0.020; 0.031) | 0.7 | 0.9 |
| Disposition index  (pmol/l·min) (umol/kg/min) | -0.001  (-0.022; 0.020) | 0.9 | -0.001  (-0.022; 0.020) | 0.9 | 0.99 |
| *CDC123* **rs12779790/**  Minor G risk-allele |  |  |  |  |  |
| M value (umol/kg/min) | 0.016  (-0.02; 0.052) | 0.4 | 0.016  (-0.02; 0.052) | 0.4 | 0.8 |
| 1^st^ phase insulin secretion  pmol/min | 0.045  (-0.034; 0.123) | 0.3 | 0.047  (-0.041; 0.134) | 0.3 | 0.3 |
| 2^nd^ phase insulin secretion  pmol/min | -0.003  (-0.071; 0.02460) | 0.9 | 0.006  (-0.091; 0.1042) | 0.9 | 0.2 |
